# Complete Genome Sequence of a Polyomavirus Recovered from a Pomona Leaf-Nosed Bat (*Hipposideros pomona*) Metagenome Data Set

**DOI:** 10.1128/genomeA.01053-16

**Published:** 2017-01-19

**Authors:** Paul G. Cantalupo, Christopher B. Buck, James M. Pipas

**Affiliations:** a Department of Biological Sciences, University of Pittsburgh, Pittsburgh, Pennsylvania, USA; b Lab of Cellular Oncology, National Cancer Institute, Bethesda, Maryland, USA

## Abstract

We report here the complete genome sequence of a polyomavirus found in a nasal/rectal metagenome of *Hipposideros pomona* (Pomona leaf-nosed bat). Interestingly, the genetic organization and phylogenetic relationships of the new virus suggest greater similarity to recently discovered fish-associated polyomaviruses rather than to polyomavirus species previously observed in bats.

## GENOME ANNOUNCEMENT

Polyomaviruses are nonenveloped icosahedral viruses with a circular double-stranded DNA (dsDNA) genome about 5 kb in size and known to infect mammals and birds. Many new polyomaviruses have been discovered since the advent of next-generation sequencing technologies, and polyomavirus sequence detection has been extended to several fish and arthropod species (1–3). Polyomavirus genomes are divided into early and late-gene regions, which occupy opposing strands. The 5′ ends of the early and late regions are separated by a regulatory region (RR) containing the origin of replication. Alternatively spliced early region transcripts encode various tumor (T) antigens, including large T (LTAg) and small T, while the major and minor capsid proteins (VP1 and VP2, respectively) are encoded in the late region.

We applied our custom viral discovery pipeline to the metagenomes of Chinese bats (4) and found a previously unidentified 4,398-bp contig from *Hipposideros pomona* (SRR2063883 library). A BLASTX search showed 41% identity between the contig and the VP1 protein of black sea bass polyomavirus (BassPyV1; accession no. NC_025790). Using an in-house script, a second contig was used to construct a complete circular sequence of 4,676 bp. The complete genome of the Pomona leaf-nosed bat-associated polyomavirus 1 (PomBAPyV1) was covered at a read depth from 9 to 502×, with 9,236 total reads.

PomBAPyV1 contains a typical opposing arrangement of open reading frames (ORFs) containing LTAg, VP1, and VP2 genes. Similar to fish-associated polyomaviruses, LTAg appears to be encoded by a single nonspliced ORF. A BLASTP search using the predicted LTAg protein sequence of PomBAPyV1 showed BassPyV1 LTAg as the top hit, with 37% identity. Most polyomavirus LTAgs contain an HPDKGG motif that is a hallmark of DNAJ chaperone protein homologs. PomBAPyV1 LTAg encodes an HPDKDP motif, and an HHpred search (5) confirms predicted structural similarity to DNAJ homologs, including the DNAJ domain of SV40 LTAg (*P* value, 1.7e-7). A canonical LXCXE motif that might mediate interactions with the retinoblastoma (Rb) family of tumor suppressor proteins was found near the carboxy-terminal end of LTAg. A characteristic nucleotide binding motif (GPFDCGK) was observed at LTAg residues 376 to 382. No ORFs associated with small T, middle T, or alternative to large T open reading frame (ALTO) were found, again suggesting an organization similar to the fish-associated polyomaviruses (1).

Phylogenetic analyses of the predicted 348-amino acid (aa) VP1 protein indicate that it occupies the same clade as VP1 proteins found in fish-associated polyomaviruses (bootstrap, 96%) (6). The predicted 511-aa VP2 protein encodes a predicted N-terminal myristoylation sequence but otherwise shows little primary sequence similarity to previously reported VP2 proteins. The only significant polyomavirus-related hit in a DELTA-BLAST search using VP2 was BassPyV1 VP2, with 22% identity and an *E* value of 2e-10.

The genomic organization and phylogenetics of PomBAPyV1 indicate that it is most closely related to polyomaviruses previously observed in fish. It remains unclear whether the source of the virus is dietary/environmental or reflects productive infection of the bat itself.

### Accession number(s).

The genome sequence was deposited in ENA at LT605004.
